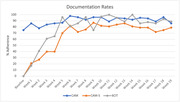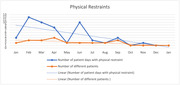# Reducing the Use of Physical Restraints in the care of Persons with Delirium and Dementia: A Quality Improvement Project

**DOI:** 10.1002/alz.093335

**Published:** 2025-01-09

**Authors:** Elizabeth Reaves Houston, Kimberly Mournighan, David H Lynch

**Affiliations:** ^1^ UNC Chapel Hill, Chapel Hill, NC USA

## Abstract

**Background:**

Hospitalized older adults, especially those with Alzheimer’s Disease and Related Dementias (PwD), are at high risk for delirium and distressing behaviors. Using physical restraints leads to functional decline and increased mortality. Our project aims to reduce restraint use by implementing a 4Ms approach for enhanced delirium management.

**Methods:**

Our interdisciplinary team used the Plan‐Do‐Study‐Act methodology over a 7‐month period (including 8 weeks of training) to introduce the 4Ms (Mentation, Mobility, Medication, what Matters) model in our 25‐bed Acute Care for Elders (ACE) unit. Nurses were trained in the Confusion Assessment Method (CAM) for delirium assessment, CAM‐S for delirium severity, and Six‐item Cognitive Impairment Test (6CIT) for cognitive impairment. Staff conducted the 6CIT per admission and CAM/CAM‐S per shift for patients aged ≥65, with electronic score documentation. Physical therapy assessed mobility within 24 hours of admission and during medical rounds. Medication reviews, led by the medical team and a geriatric pharmacist, occurred for new admissions. What Matters considerations were addressed through comprehensive geriatric assessments. Chart review collected data. An electronic record quantified physical restraint orders for six months pre‐ and post‐intervention, by patient days and distinct patients per month.

**Results:**

In the 19 weeks following data collection initiation, ACE unit patients (n = 362) averaged 81 years old, 60% female, 83% white, 14% Black, 18% were PwD, and 24% had significant cognitive impairment (6CIT score). Twenty‐two percent experienced incident delirium during a mean 6‐day stay. Documentation improved: CAM (68% to 86%), CAM‐S (0% to 79%), 6CIT (0% to 89%). A 4Ms checklist achieved a 96% completion rate with 37.5% receiving a geriatric assessment, 25% were discharged with an ADL disability, and 12.5% had medication deprescribed. Restraint use decreased from 7.7 patient days per month to 2.2, distinct patients with restraints decreased from 2.7 to 1.7.

**Conclusions:**

In a high‐risk older adult population, a low‐resource quality improvement intervention effectively implemented the 4Ms model of care, showing a positive trend in reducing restraint use.